# 4240 The relationship between family structure and quality of life among children with asthma

**DOI:** 10.1017/cts.2020.283

**Published:** 2020-07-29

**Authors:** Magda Shaheen, Arneshia LA’Shelle Bryant-Horn, Senait Teklehaimanot

**Affiliations:** 1 David Geffen School of Medicine at UCLA; 2 Charles R Drew University

## Abstract

OBJECTIVES/GOALS: Asthma is a life-long, chronic lung disease that inflames and narrows the airways. Its effects on quality of life in children can be exacerbated. The goal of this study was to investigate the link between asthma, family structure and demographics and how it impacts quality of life in children. METHODS/STUDY POPULATION: We analyzed data from a cross sectional study of the 2016-2017 National Survey of Children’s Health, NSCH, to assess the relationship between determinants of health variables and the outcome variable of parent’s report of child’s perceived health status (quality of life). The study population was children under the age of 18. Data were analyzed using descriptive, bivariate analysis using Chi square, and multiple logistic regression of quality of life and family structure adjusting for confounding variables. RESULTS/ANTICIPATED RESULTS: The study included 5,687 children. Significant predictors of asthmatic children’s quality of life were severity of asthma, self-perceived mental/physical health status of adults, neighborhood safety for children (p <0.05). The interaction between family structure and asthma severity was significant indicating that asthma severity was an effect modifier. Among children with mild asthma, predictors of quality of life were self-perceived mental/physical health status of adults in the household, neighborhood safety of children, physical activity status of children (p<0.05). Among children with severe asthma, predictors were family structure and physical/mental health of adults (p<0.05). DISCUSSION/SIGNIFICANCE OF IMPACT: This study suggests children with severe asthma who are born to single mothers with lower parental reporting of physical/mental health status had a lower quality of life. A longitudinal study could be implemented to target these three measures to improve quality of life among these children. Also, a culturally adapted intervention involving community, parents, and providers is needed to improve the quality of life of the children with asthma.

